# ICTV Virus Taxonomy Profile: Botourmiaviridae 2024

**DOI:** 10.1099/jgv.0.002047

**Published:** 2024-11-21

**Authors:** Livia Donaire, Jiatao Xie, Luca Nerva, Daohong Jiang, Shin-Yi Lee Marzano, Sead Sabanadzovic, Massimo Turina, María A. Ayllón

**Affiliations:** 1 Biology of Stress and Plant Pathology Department, CEBAS-CSIC, Murcia, Spain; 2 Plant Pathology Department, College of Plant Science and Technology, Huazhong Agricultural University, Wuhan, PR China; 3 CREA – Research Centre for Viticulture and Enology, Congegliano, Italy; 4 USDA-ARS, Toledo, OH, USA; 5 Department of Agricultural Science and Plant Protection, Mississippi State University, Mississippi, MS, USA; 6 Institute for Sustainable Plant Protection – CNR, Torino, Italy; 7 Centro de Biotecnología y Genómica de Plantas, CBGP (UPM-INIA/CSIC), and Dpto. Biotecnología-Biología Vegetal, ETSIAAB, Universidad Politécnica de Madrid (UPM), Madrid, Spain

**Keywords:** *Botourmiaviridae*, ICTV report, taxonomy

## Abstract

The family *Botourmiaviridae* includes viruses with a mono- or multi-segmented positive-sense RNA genome that infect plants and filamentous fungi. The family includes the genera *Ourmiavirus* (plant viruses), *Botoulivirus*, *Betabotoulivirus*, *Magoulivirus*, *Scleroulivirus*, *Betascleroulivirus*, *Gammascleroulivirus*, *Deltascleroulivirus*, *Epsilonscleroulivirus*, *Penoulivirus*, *Rhizoulivirus* and *Betarhizoulivirus* (fungal viruses). This summary is based on the International Committee on Taxonomy of Viruses (ICTV) Report on the family *Botourmiaviridae*, which is available at ictv.global/report/botourmiaviridae.

## Virion

**Fig. 1.**
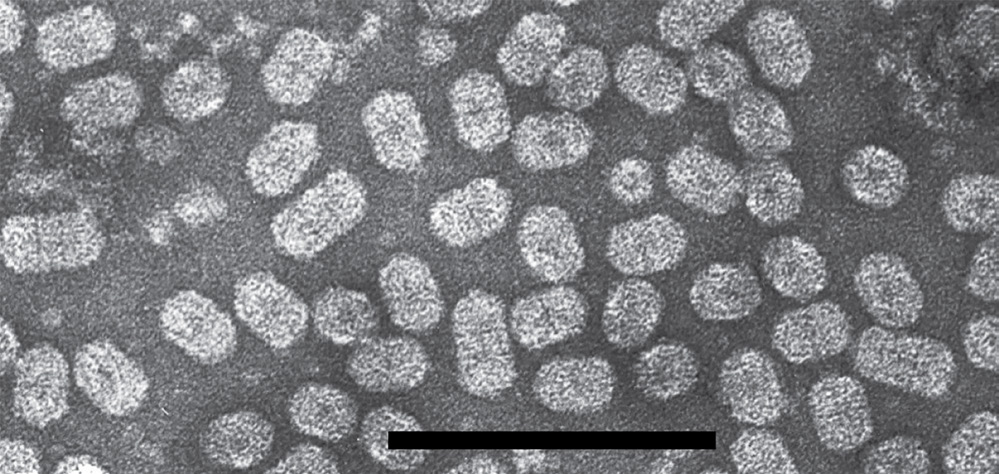
Virion morphology. Negative-contrast electron micrograph (uranyl acetate) of purified particles of Ourmia melon virus (bar, 100 nm).

Members of the genus *Ourmiavirus* are plant viruses with non-enveloped bacilliform virions composed of a single 23.8 kDa coat protein. Electron microscopy reveals particles with conical ends (apparently hemi-icosahedral) and cylindrical bodies that are 18 nm in diameter (Table 1, Fig. 1). Most particles consist of two discs (giving a particle length of 30 nm), while others have three (37 nm) or, more rarely, four (45.5 nm) or six discs (62 nm). Members of other genera are non-encapsidated mycoviruses.

**Table 1.** Characteristics of members of the family *Botourmiaviridae*

Example	Ourmia melon virus VE9 (RNA1: EU770623; RNA2: EU770624; RNA3: EU770625), species *Ourmia melon virus*, genus *Ourmiavirus*
Virion	Bacilliform (18×30–62 nm) with a 23.8 kDa CP (*Ourmiavirus*) or non-encapsidated (members of other genera)
Genome	Positive-sense, mono-segmented RNA of approximately 2–5 kb (members of 11 genera) or tri-segmented RNA genome of 2.8, 1.1 and 0.97 kb (*Ourmiavirus*)
Replication	Cytoplasmic; virion assembly is coupled to active replication (*Ourmiavirus*)
Translation	From genomic RNA; each genomic segment is monocistronic
Host range	Plants (*Ourmiavirus* only) and fungi
Taxonomy	Realm *Riboviria*, kingdom *Orthornavirae*, phylum *Lenarviricota*, class *Miaviricetes*, order *Ourlivirales*, family *Botourmiaviridae*; >10 genera with >150 species

## Genome

**Fig. 2.**
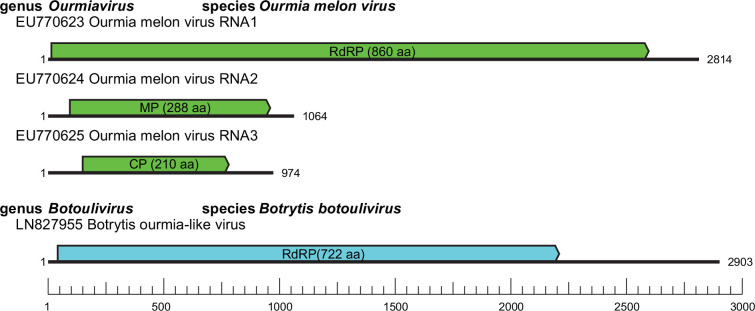
Genome organization of representative isolates of the family *Botourmiaviridae*.

The genome of ourmiaviruses consists of three segments of positive-sense RNA (2814, 1064 and 974 nt for Ourmia melon virus) [1], encoding an RNA-directed RNA polymerase (RdRP, 97.5 kDa; RNA1), a movement protein (MP, 31.6 kDa; RNA2) and a coat protein (CP, 23.8 kDa; RNA3) (Fig. 2) [2]. Members of genera other than *Ourmiavirus* have a single genome segment of 1671–5234 nt encoding an RdRP [3,5]. Unlike other members of the family, the genomes of the magoulivirus, Magnaporthe oryzae ourmia-like virus 1; the scleroulivirus, Magnaporthe oryzae botourmiavirus 9 and the gammascleroulivirus, Magnaporthe oryzae botourmiavirus 6 are polyadenylated at the 3′-end [6,7].

## Replication

Ourmiavirus replication is dependent on the viral RdRP. The synthesis of the ourmiavirus CP from actively replicating RNA3 is necessary for both virion assembly and systemic infection of the host [2]. The ourmiavirus MP determines symptoms and forms tubular structures involved in cell-to-cell movement [8] and may undergo post-translational modification. Replication of mycoviruses (genera other than *Ourmiavirus*) is strictly dependent on the virus RdRP.

## Pathogenicity

Members of the genus *Ourmiavirus* infect plants; Ourmia melon virus infects melon, producing chlorotic spots and irregular ringspots [9]; Epirus cherry virus produces rasp-leaf symptoms in cherry and cassava virus C induces severe stunting and a yellow mosaic pattern in cassava. Members of the other genera infect fungi; their previously reported association with oomycetes [10] is unlikely since their presence has not been confirmed in isolates of vast collections of cultivable oomycetes.

## Taxonomy

Current taxonomy: www.ictv.global/taxonomy. Ourmiaviruses are clearly separated from members of the other genera based on their host and the number of genomic segments. For other botourmiaviruses, members of different genera are <70% identical in their complete RdRP amino acid sequences. Binomial names for all species will be introduced next year.

## Resources

Full ICTV Report on the family *Botourmiaviridae*: ictv.global/report/botourmiaviridae.

## Abbreviations

CP: coat protein
ICTV: International Committee on Taxonomy of Viruses
MP: movement protein
RdRP: RNA-directed RNA polymerase
